# Artificial Intelligence in Sports Cardiology: Advancing Cardiovascular Screening and Diagnosis

**DOI:** 10.7759/cureus.104174

**Published:** 2026-02-24

**Authors:** Khalil Jalkh, Adnan AlJaroudi, Wael Aljaroudi, Haitham Hreibe

**Affiliations:** 1 Cardiology, Medical College of Georgia, Augusta University, Augusta, USA; 2 High School, Lakeside High School, Evans, USA

**Keywords:** artificial intelligence in health care, athlete cardiovascular screening, echocardiography, electrocardiography, sports cardiology, sudden cardiac death (scd)

## Abstract

Sudden cardiac death in athletes, though uncommon, remains a major concern in sports cardiology. Many responsible cardiovascular conditions, including cardiomyopathies, inherited channelopathies, valvular disease, and congenital coronary anomalies, may remain asymptomatic until intense physical exertion. Current pre-participation screening relies on clinical history, physical examination, electrocardiography, and selective cardiac imaging. While effective, these tools are limited by interobserver variability, dependence on specialist expertise, and difficulty distinguishing physiological athletic remodeling from pathological disease. These limitations have prompted growing interest in artificial intelligence (AI) as an adjunct to cardiovascular screening in athletes.

This review summarizes current evidence on AI applications in sports cardiology, with a focus on electrocardiography, digital auscultation, transthoracic echocardiography, and selected imaging modalities. AI-enhanced electrocardiographic analysis has demonstrated improved sensitivity compared with traditional criteria for detecting left ventricular hypertrophy, long QT syndrome, including concealed forms, Brugada syndrome, electrolyte abnormalities, and aortic stenosis. Several deep learning models identify disease patterns even when conventional electrocardiographic parameters appear normal, addressing a key limitation of standard screening approaches.

AI-assisted digital auscultation improves the detection of pathological murmurs and differentiation from benign flow murmurs, supporting earlier identification of valvular disease. In echocardiography, AI-guided image acquisition and automated analysis improve access, workflow efficiency, and measurement consistency, with diagnostic performance approaching expert interpretation.

This review proposes a pragmatic AI-integrated screening framework that complements clinician judgment rather than replacing it. Although promising, limitations remain, including limited athlete-specific training data, false-positive risk related to physiological remodeling, and the need for external validation. When thoughtfully integrated into clinical workflows, AI may enhance early detection of occult cardiac disease and improve cardiovascular risk stratification in athletes.

## Introduction and background

Athletes are often seen as the epitome of peak physical health, yet they remain vulnerable to serious cardiac conditions that can lead to sudden cardiac arrest. Fabrice Muamba, a 23-year-old midfielder for Bolton Wanderers, serves as a striking example of this reality. On March 17, 2012, during an FA Cup quarter-final against Tottenham Hotspur, Muamba collapsed on the field despite no prior cardiac history or symptoms. Fortunately, immediate resuscitation efforts were performed, restoring circulation after a 78-minute circulatory arrest. His case underscores the importance of screening and early diagnosis of potentially life-threatening conditions such as hypertrophic cardiomyopathy, arrhythmogenic right ventricular cardiomyopathy, or channelopathies in triggering life-threatening arrhythmias during intense exertion. His ordeal catalyzed improvements in pre-participation cardiac screening and reinforced the need for heightened awareness and preparedness to prevent sudden cardiac death (SCD) in athletes.

The incidence of SCD in competitive athletes is estimated at approximately one in 50,000 athlete-years, with higher rates reported among male athletes and those participating in high-intensity sports [1,2].

But it is not just about rare, high-profile cases. Everyday athletes might ignore warning signs like palpitations, dizziness, or unexplained shortness of breath, as they would attribute them to pushing their limits. The culture of "no pain, no gain" can be dangerously misleading when it comes to heart health. This further emphasizes the importance of preventive measures. Electrocardiograms and echocardiograms can detect abnormalities early, which has led some countries and athletic governing bodies mandate pretesting for competitive athletes [3].

In the athletic population, key cardiovascular pathologies warranting screening include hypertrophic cardiomyopathy, inherited channelopathies such as long QT syndrome and Brugada syndrome, aortic stenosis, congenital coronary artery anomalies, and coronary artery disease. Common screening tools include ECG and echocardiography, with advanced imaging reserved for selected cases. While these tools are effective, their sensitivity in reliably excluding these conditions depends a lot on the interpreter. Given that screening is often performed by primary care physicians rather than specialized cardiologists, the potential for diagnostic uncertainty is increased. Artificial intelligence (AI) offers a promising solution by enhancing diagnostic accuracy through advanced pattern recognition and predictive analytics, leveraging vast datasets to improve the interpretation of screening results and reduce the risk of missed diagnoses.

## Review

Methods

A structured literature search was performed to identify relevant studies examining the application of AI in screening and diagnostic pathways within sports cardiology. Searches were conducted across three major databases (MEDLINE, PubMed, and Embase) to ensure broad coverage. Search terms included combinations of concepts such as “sports cardiology” with “artificial intelligence”, “machine learning”, “deep learning”, or “neural networks”; AI-based electrocardiographic assessment of left ventricular hypertrophy, channelopathies, and electrolyte abnormalities; AI-assisted echocardiographic screening; AI applications in cardiac CT imaging among athletes; and the use of AI-enabled wearable technologies in athletic populations.

While a systematic search strategy was employed to comprehensively capture relevant literature, this review is narrative in nature. Identified studies were screened for relevance, grouped into thematic domains, and selectively synthesized to provide a conceptual and clinically focused overview of current and emerging AI applications in sports cardiology, rather than a formal systematic review or meta-analysis.

AI-assisted ECG analysis

Across ECG-based applications in sports cardiology, convolutional neural networks (CNNs) represent the most frequently utilized deep learning architecture, with most studies relying on supervised models trained on raw waveform data.

AI-Assisted ECG for the Detection of Left Ventricular Hypertrophy

Earlier work on automated ECG interpretation has been around for decades, but most of those systems used rule‐based or heuristic methods rather than modern AI/machine learning (ML) techniques we see today. The earliest attempt at automating the detection of left ventricular hypertrophy (LVH) on ECG was done by Pipberger et al. [4] in 1975 and later by Swartz et al. [5] (creating GE’s Marquette 12SL program) in 1982. While the term "artificial intelligence" was not as prevalent as it is today, these efforts laid the groundwork for the application of computer algorithms in cardiac diagnostics. The shift toward self-learning systems, such as neural networks, in the mid-1990s and with Heden et al.’s work representing one of the earliest AI-driven efforts for ECG interpretation [6], research on AI interpretation of ECGs had persisted but relatively slowed down till 2019. The endeavor was rejuvenated after a research team from Google published its groundbreaking research on AI in 2017 [7].

A recent meta-analysis published in Nature in July 2024 by Siranart et al. [8] evaluated the diagnostic performance of AI in detecting LVH using ECG, benchmarking it against traditional criteria like Sokolow-Lyon and Cornell indices. The study systematically reviewed eight observational studies (including one case-control, five retrospective, and two prospective cohort studies), comprising a total of 66,479 participants (14,190 with LVH and 52,289 without).

All the AI models used a combination of ECG waveforms, patient demographics, and clinical information to better detect LVH. The results were impressive, as AI-based methods achieved an overall accuracy (measured by the area under the curve) of 0.87. This performance surpassed traditional ECG criteria, such as the Sokolow-Lyon (0.68) and Cornell criteria (0.60). The AI approach had a sensitivity of 69% and specificity of 87%, whereas the traditional methods had much lower sensitivities (25% for Sokolow-Lyon and 19% for Cornell), despite slightly higher specificities (92% and 94%, respectively). These findings highlight AI’s significant potential to enhance LVH detection through improved sensitivity, marking it as a promising tool for more effective cardiovascular screening in athletes. Overall, AI-assisted ECG models for LVH detection have demonstrated diagnostic performance with area under the receiver operating characteristic curve (AUROC) values generally ranging from approximately 0.85-0.90 across studied cohorts, consistently outperforming traditional voltage-based criteria.

Key studies evaluating AI-assisted ECG interpretation for the detection of LVH are summarized in Table 1.

**Table 1:** Table summarizing relevant articles on AI-assisted interpretation of ECGs for detecting LVH, which contributed to this review. LVH: left ventricular hypertrophy; LV: left ventricular; CMR: cardiac magnetic resonance; CNN: convolutional neural network; DNN: deep neural network; MESA: Multi‐Ethnic Study of Atherosclerosis; LSTM: long short-term memory network; ENN: ensemble neural network; NN: neural network; BART: Bayesian additive regression trees; ECG: electrocardiogram; Echo: echocardiography; MRI: magnetic resonance imaging; AUC: area under the curve; AUROC: area under the receiver operating characteristic curve; UKB: UK Biobank.

Date	Authors	Type of paper	Type of AI	Number of participants	Review of outcome
July 2024	Siranart et al. [8]	Systematic review & meta-analysis	Various (majority neural networks – CNN, ENN, back-propagation NN; also BART and C5.0)​	8 studies (N = 66,479)​	Pooled analysis showed AI ECG models significantly improved LVH diagnostic accuracy over conventional ECG criteria. Summary: AUROC for AI was 0.87 (sensitivity = 69%, specificity = 87%) vs. 0.68 for Sokolow–Lyon criteria.​ AI-based ECG interpretation offers higher sensitivity than voltage criteria, though further research on clinical impact is needed.
April 2024	Cai et al. [9]	Original research	Deep learning (2D CNN with “one-shot” ECG image encoding)	42,127 ECG–Echo pairs​	Developed a 2D-CNN model that converts each 12-lead ECG into a single combined image for analysis. The 2D-CNN achieved an AUROC of 0.92 for LVH detection, outperforming logistic regression, random forest, and a prior 1D-CNN approach on the same data. Including/adding patient metadata further raised AUROC to 0.921. The model significantly outperformed traditional ECG criteria (which had an AUROC of 0.60–0.62), indicating robust improvement in sensitivity and overall accuracy.
March 2023	Dwivedi et al. [10]	Original research	Machine learning (random forest models) and deep neural network (ResNet-based CNN)	Approx. 1,000,000 ECGs (training), 250,000 (testing)​ from the Mayo Clinic database	Investigated limb-lead-only ECG models for LVH. A deep neural network using only 6 limb leads achieved an AUC of 0.92, compared to 0.98 with a full 12-lead model. Simpler random forest models had lower performance (AUC = 0.78–0.83). The AI model considerably improved sensitivity while using fewer leads, suggesting potential for easier screening (e.g., with wearable or handheld ECGs) without much loss in accuracy.
February 2023	Liu CW et al. [11]	Original research	Back-propagation neural network (feed-forward NN using engineered ECG features)	173 LVH patients (Taiwanese cohort; ECG vs. echo)​	Extracted 24 features (R/S amplitudes from 12 leads) and trained a back-propagation neural network to classify LVH (echo-confirmed) vs. non-LVH. The model attained 96.1% accuracy (sensitivity = 96.6%, specificity = 95.6%) on the test set, greatly outperforming seven conventional ECG criteria and previous AI models on the same data. The high accuracy demonstrates that a properly trained neural network can detect subtle ECG patterns of LVH that criteria-based methods miss.
August 2022	Liu CM et al. [12]	Original research	Deep learning (12-lead ECG CNN)	ECG recordings from 28,745 (for development of model), plus internal test and external validation (225 patients)​	A 12-lead ECG deep learning model was trained with LVH labels from echocardiography. It achieved an AUC of 0.89 for LVH detection (sensitivity = 90%, specificity = 69%), significantly better than cardiologists using conventional criteria (AUC = 0.64). Performance remained high in an independent cohort (AUC = 0.86)​ and an external Japanese validation set (AUC = 0.83). Notably, AI-predicted LVH also correlated with higher risk of 6-year cardiovascular and all-cause mortality (HR = 1.5–1.9), suggesting the model’s predictions carry prognostic value.
September 2022	Kokubo et al. [13]	Original research	Deep learning (CNN) vs. machine learning (logistic regression, random forest)	Data from 18,954​ patients (hospital ECG + Echo dataset)	Compared a deep learning model to traditional ML and voltage criteria for detecting LV dilation and LVH. For LVH, the CNN had an AUROC of 0.784, significantly higher than logistic regression (0.758), random forest (0.716), and conventional ECG criteria (Cornell, Sokolow–Lyon). The deep model’s sensitivity and overall accuracy were superior (p<0.001). This study reinforced that end-to-end deep learning on raw ECG outperforms both manual criteria and simpler ML models in LVH screening.
August 2022	Zhao et al. [14]	Original research	Deep learning (CNN–LSTM hybrid)	ECGs from 1,863 patients (development) + 453 (internal testing)​	Developed a 16-layer CNN-LSTM network for LVH detection from 12-lead ECG. The model achieved modest accuracy (AUC = 0.62, sensitivity = 68%, specificity = 57%) on the test set. This was an improvement over traditional criteria (Cornell AUC = 0.57, Sokolow-Lyon = 0.51), which had very low sensitivity (14–48%). The AI model’s sensitivity was higher, especially in male patients, suggesting it could serve as a better screening tool than voltage criteria, despite only moderate AUC.
June 2021	Khurshid et al. [15]	Original research	Deep learning (CNN for LV mass regression)	32,239 who underwent CMR and ECGs (training); external test sets of 4,903 + 1,371; outcomes cohort of 28,612​	Used 12-lead ECGs from the UK Biobank to train a CNN to predict left ventricular mass (LVM) as measured by cardiac MRI (termed “LVM-AI”). The CNN’s ECG-estimated LV mass correlated well with true MRI mass (r = 0.79 in the UKB test). When classifying LVH, the AI had similar discrimination to traditional ECG criteria in one cohort and better performance in another (c-statistic = 0.62 vs. 0.59). Importantly, AI-predicted LVH was associated with a higher incidence of atrial fibrillation, myocardial infarction, heart failure, and ventricular arrhythmias during follow-up. This highlights AI-ECG’s potential for improved detection and risk stratification.
March 2020	Kwon et al. [16]	Original research	Ensemble neural network (ensemble of CNN and DNN)	21,286​ patients (retrospective multi-hospital ECG + Echo)	Trained an ensemble neural network (ENN) to detect LVH from ECG. The AI achieved AUCs of 0.880 (internal validation) and 0.868 (external validation), outperforming cardiologists’ assessments and standard criteria. At equal specificity, the ENN’s sensitivity was 1.6-1.8 times higher than that of a cardiologist (using Romhilt-Estes) or Sokolow-Lyon. The study demonstrated that an AI model can substantially boost sensitivity while maintaining specificity, greatly improving upon the 30% sensitivity of traditional ECG-LVH interpretation.
May 2020	De la Garza-Salazar et al. [17]	Original research	Machine learning (C5.0 decision tree with logistic regression features)	432​ patients (ECG vs. Echo)	Applied a C5.0 decision-tree algorithm to ECG measurements. The resulting 5-level decision tree used 6 key ECG features and achieved (in two different groups) 71-73% accuracy (sensitivity = 80%, specificity = 53-69%) on internal and external validation sets. The ML-driven approach significantly increased sensitivity while maintaining moderate specificity, indicating an overall better diagnostic yield than classic criteria.
March 2019	Sparapani et al. [18]	Original research	Bayesian additive regression trees (BART) ensemble	4,714 patients​ (MESA cohort; training 3,774, validation 940)	Developed a BART-based model to estimate LV mass from ECG and identify LVH (using MRI as the gold standard). In the validation set, BART-LVH criteria showed higher sensitivity (29%) than traditional voltage criteria (e.g., Sokolow-Lyon = 21.7%, Cornell = 5-15%) at similar high specificity (>93%). BART-predicted LVH was also strongly associated with outcomes over 12.3 years, predicting a higher risk of death and cardiovascular events (hazard ratios = 1.5-1.9, similar to MRI-defined LVH). Conclusions: the BART AI model provided superior diagnostic and prognostic performance compared to standard ECG criteria, approaching the fidelity of cardiac MRI for LVH detection.

AI-Assisted ECG for the Detection of Channelopathies/Long QT

Long QT syndrome (LQTS), a cardiac ion channelopathy associated with life-threatening arrhythmias, poses significant diagnostic challenges due to its heterogeneous presentation. While traditional diagnosis relies on ECG parameters like the corrected QT (QTc) interval and genetic testing, up to 30% of patients exhibit normal QTc values (concealed LQTS), complicating identification. Recent advancements in AI, particularly machine learning (ML) and deep learning (DL), have shown promise in improving LQTS detection and subtype classification.

A recent meta-analysis of eight studies reported a pooled sensitivity and specificity of 0.87 and 0.91, respectively, with an AUROC of 0.95 for detecting LQTS [19]. These models outperformed traditional QTc-based assessments, particularly in cases of concealed LQTS, by effectively analyzing T-wave morphology and depolarization patterns. Similarly, a recent multicenter study analyzed 4,521 ECGs from 990 patients. Based on average QTc intervals, the model identified patients with congenital LQTS, achieving an overall area under the curve (AUC) of 0.74 (95% CI, 0.69-0.78) [20]. The model demonstrated improved performance in distinguishing LQTS, with an overall AUC of 0.81 (95% CI, 0.80-0.85) across all validation datasets. Notably, the model maintained strong diagnostic performance in detecting concealed LQTS, achieving an AUC of 0.78 (95% CI, 0.72-0.83) [20]. AI models consistently demonstrated diagnostic accuracy across different studies, even in patients with normal QTc intervals, marking a significant advancement for early screening.

Genotype prediction and subtype differentiation: AI shows potential in predicting LQTS genotypes (LQT1, LQT2, LQT3). Wu et al.’s subgroup analysis revealed higher accuracy for LQT2 (AUROC: 0.92) than LQT1 (0.89), likely due to distinct T-wave abnormalities usually found in LQT2 [19]. Jiang et al.’s model differentiated LQT1 and LQT2 with AUROCs of 0.95 in the internal validation model (with the balanced sensitivity and specificity at 0.90 and 0.79, respectively, and a positive predictive value (PPV) of 0.69) and 0.91 in the external validation model (with the balanced sensitivity and specificity at 0.82 and 0.88, respectively, and a PPV of 0.77) [20]. However, the model’s performance declined for LQT3, likely due to limited data availability for this subtype.

A study by Aufiero et al. utilized a DL model to identify genotype-positive LQTS patients. In that study, the DL models again outperformed conventional QTc measurements by matching the sensitivity of an expert cardiologist while demonstrating higher specificity. To shed light on how the model obtained higher specificity, the study incorporated an explainable AI approach to better understand the model’s decision-making process. This analysis revealed that the model primarily relied on the onset of the QRS complex for classification. This was a surprising finding, as it shifts attention from the traditionally emphasized repolarization phase and T-wave abnormalities, which have long been central to LQTS pathophysiology. Instead, it suggests potential disruptions in early ventricular depolarization in individuals with LQTS-related genetic mutations. This observation opens new insights for understanding LQTS, indicating that ion channels involved in depolarization, alongside or beyond potassium channels, may play a significant role in the disease process. These findings suggest that AI could help guide targeted genetic testing and personalized therapies, although validation in larger, more diverse cohorts is needed, particularly for rare LQTS subtypes [21].

Despite promising results, several limitations impede clinical translation. First, most models lack external validation. Second, datasets are biased, given that the papers are predominantly European cohorts, which limits generalizability, as LQTS genetic variants vary ethnically. Third, interpretability remains a hurdle. While explainable AI techniques linked decisions to QRS complexes [21], most models operate as “black boxes,” hindering clinician trust. Additionally, small sample sizes for LQT3 and drug-induced LQTS restrict model utility. Across studies, AI-assisted ECG models for LQTS detection have demonstrated AUROC values generally ranging from approximately 0.74 to 0.95, with preserved diagnostic performance even in patients with concealed phenotypes.

Key studies examining AI-assisted ECG analysis for the detection of LQTS and other channelopathies are provided in Table 2.

**Table 2:** Table summarizing relevant articles on AI-assisted interpretation of ECGs for detecting long QT syndrome (LQTS) and other channelopathies, which contributed to this review. LQTS: long QT syndrome; cLQTS: congenital long-QT syndrome; TdP: torsade de pointes; diTdP: drug-induced torsade de pointes; BrS: Brugada syndrome; SQTS: short QT syndrome; QTc: corrected QT interval; AUC: area under the receiver operating characteristic curve; CNN: convolutional neural network; DNN: deep neural network; ECG: electrocardiogram; ICD: implantable cardioverter-defibrillator.

Date	Authors	Type of article	Type of AI used	Patients	Outcome summary
February 2025	Bos et al. [22]	Original research	Deep neural network (CNN)	1599 total patients, 808 showing QTc prolonged (2,987 ECGs). Control group = 361,069 patients (989,313 ECGs)	AI-ECG model distinguished congenital LQTS from acquired (drug-induced) QT prolongation with AUC ≈0.896 (85% accuracy, sensitivity 77%, specificity 87%). In patients with extreme QT prolongation (QTc >99th percentile), the AI model functioned as a “mutation detector” to identify those with an LQTS-causing gene mutation rather than an acquired cause.
March 2024	Dehkordi et al. [23]	Review	Various (deep learning focus)	N/A (review)	Comprehensive review of AI in LQTS diagnosis. Concluded that deep learning ECG algorithms can outperform the QTc in detecting LQTS (including concealed cases)​ and even predict genotype (LQT1 vs. LQT2).
April 2024	Jiang et al. [20]	Original research	Convolutional neural network	990 patients (4521 ECG)	A deep learning model using 12-lead ECGs for LQTS achieved an AUC of 0.93 for detecting LQTS and an AUC of 0.91 for distinguishing genotypes (LQT1 vs. LQT2) in an external cohort. It significantly outperformed expert QTc measurements, notably in identifying cases with normal/borderline QTc (concealed LQTS) that QTc alone missed.
October 2024	Rudic et al. [24]	Case report	Smartwatch ECG (AI algorithm)	1	Describes a 39-year-old male whose smartwatch ECG (Withings ScanWatch) repeatedly showed QT = 500 ms, prompting clinical evaluation. The AI-enabled wearable reliably measured QT intervals​ and facilitated the diagnosis of congenital LQTS (confirmed KCNH2 gene mutation), which had been previously unrecognized. This case illustrates how consumer devices with AI can aid in the early detection of LQTS.
November 2023	Pasero et al. [25]	Original research	Neural networks (“shallow”)	104 patients, with 37 of them having a prior arrhythmic event.	Machine learning algorithms (shallow neural networks) identified ECG patterns distinguishing SQTS patients less likely to experience life-threatening arrhythmias. This AI-based risk stratification could help pinpoint which SQTS patients might not require aggressive interventions (ICDs, etc.).
November 2023	Melo et al. [26]	Original research	Deep neural network	1455 consecutive patients going for an electrophysiology study. Training set = 1154 ECGs. Validation set = 370 ECGs.	Developed a deep learning algorithm to detect the hidden ECG signature of Brugada syndrome (BrS) without drug challenge. The model achieved 88.4% accuracy (AUC = 0.934) in validation, successfully identifying BrS in cases where the diagnostic type 1 pattern was not obvious on resting ECG.
August 2022	Liao et al. [27]	Original research	Convolutional neural network	Training set = 1570 ECGs. Testing set = 1190 ECGs.	Used a deep learning model on continuous 12-lead Holter ECG recordings to improve Brugada syndrome diagnosis. The CNN achieved an AUC of 0.976 on standard 10-sec ECGs and 0.975 on 24-hour Holter data for detecting type 1 Brugada pattern, comparable to expert cardiologists. When applied to Holter recordings, the model detected spontaneous Brugada type 1 episodes in 48% of patients with previously drug-induced Brugada and 33% of those with suspected Brugada, while yielding 0% false positives in healthy controls.
May 2022	Aufiero et al. [21]	Original research	1D CNN (explainable AI)	10,000 controls (172 LQTS1, 214 LQTS2, and 72 LQTS3 patients adding up to 458 LQTS to train); 2,200 controls (32 LQTS1, and 80 LQTS2 patients adding up to 112 LQTS for external validation)	Trained an explainable 1D-CNN on a large ECG dataset to detect genotype-positive congenital LQTS. The model’s per-subtype performance: sensitivity = 84-90%, specificity = 92-96% for LQT1, LQT2, and LQT3 (AUC 0.90-0.92). It outperformed QTc measurements in identifying LQTS carriers and even outperformed an expert LQTS cardiologist in specificity​ while matching the expert’s sensitivity. Explainable AI revealed the QRS onset as a previously under-appreciated ECG region informative for LQTS detection, offering new pathophysiological insights.
February 2022	Liu et al. [28]	Original research	Deep CNN (two-stage)	276 Brugada vs. 276 controls (train); external test cohort	A two-step deep learning model was created for Brugada syndrome screening. It first learned to recognize right bundle-branch patterns, then identified the Brugada type 1 ECG. In the internal test, it reached an AUC of 0.96 (sensitivity 88.4%, specificity 89.1%), and on an independent external cohort, an AUC of 0.89 (86% sensitivity, 90% specificity). The AI’s diagnostic agreement with the gold standard was substantially higher than that of general cardiologists, suggesting it can serve as a robust Brugada screening tool.
October 2021	Prifti et al. [29]	Original research	Deep learning (ECG features)	1029 healthy subjects before and after sotalol intake (n = 14,135 ECGs); 487 cLQTS patients (n = 1083 ECGs: 560 type 1, 456 type 2, 67 type 3), 48 patients with drug-induced TdP (n = 1105 ECGs, with 147 obtained within 48 h of a diTdP episode).	A multinational study applied deep learning to ECGs for two purposes: (1) detect congenital LQTS and (2) predict the risk of drug-induced arrhythmias (sotalol). The AI algorithm identified subtle ECG alterations associated with LQTS genotypes – it detected LQT2 genotype carriers with particularly high accuracy, with decent performance for LQT1 and LQT3 as well. The model could also recognize ECG changes indicative of IKr blockade (a common mechanism of drug-induced QT prolongation), potentially differentiating high-risk individuals.
May 2021	Bos et al. [30]	Original research	Convolutional neural network	2,059 (967 LQTS, 1,092 controls)	An AI-enhanced 12-lead ECG model was trained to detect concealed LQTS. In a test of patients evaluated for LQTS, the model achieved an AUC of 0.900, significantly better than using QTc alone (AUC = 0.824). Importantly, among genotype-positive patients with normal resting QTc, the AI-ECG substantially improved detection (AUC = 0.863 vs. 0.741 for QTc). The network could also triage the probable LQTS subtype, correctly anticipating the specific genotype (LQT1, LQT2, LQT3) before genetic testing.

AI-Assisted ECG for the Detection of Electrolyte Imbalance

In professional athletes, dehydration and electrolyte imbalances are common and can significantly impact performance and cardiovascular health. Rapid screening for electrolyte disturbances holds substantial potential in sports cardiology. The integration of AI-driven ECG analysis presents a promising approach for non-invasive electrolyte monitoring. A systematic review and meta-analysis by Dasdelen et al. assessed the diagnostic accuracy of AI models in detecting electrolyte abnormalities. Their analysis encompassed data from 21 studies, incorporating over 600,000 ECGs, highlighting the potential of AI-enhanced ECG interpretation as a screening tool in athletic populations [31].

The meta-analysis highlighted several key findings, particularly in AI-based detection of potassium imbalances. AI models demonstrated moderate-to-high diagnostic capability, especially in diagnosing hyperkalemia (serum K⁺ >5.5 mmol/L), achieving a pooled sensitivity of 0.856 (95% CI: 0.829-0.879) and specificity of 0.788 (0.744-0.826), with a diagnostic odds ratio (DOR) of 21.8 (17.8-26.7). Detection of hypokalemia (K⁺ <3.5 mmol/L) had slightly lower accuracy, with a sensitivity of 0.824 (0.785-0.856), specificity of 0.724 (0.668-0.774), and a DOR of 12.27 (9.15-16.47). External validation showed a decline in specificity compared to internal testing, highlighting limitations in the model’s generalizability. Furthermore, 12-lead ECGs outperformed single-lead setups, yielding higher specificity (0.867 vs. 0.776) and a greater DOR (35.61 vs. 27.69) for hyperkalemia detection, likely due to the superior spatial resolution provided by multi-lead analysis.

Calcium and sodium imbalance studies were limited and showed lower accuracy and are not of significant diagnostic use. These imbalances posed greater diagnostic challenges due to subtler ECG manifestations compared to potassium disturbances [32,33].

Some studies attempted to interpret the AI decision-making process using gradient class activation maps, revealing that the model primarily relied on both the QRS complex and T waves for hyperkalemia detection [34,35].

Despite the growing number of studies exploring AI-assisted ECG analysis for electrolyte abnormalities, critical limitations persist. Dasdelen et al.’s meta-analysis showed that almost half of the studies had a high risk of bias in patient selection. This was primarily due to non-consecutive sampling or case-control study designs. Furthermore, the majority relied on retrospective data, with limited prospective validation across diverse populations [31]. Heterogeneity in model architectures and validation protocols further complicates cross-study comparisons, undermining real-world generalizability. While current AI models are not yet substitutes for serum electrolyte testing, their integration into wearable technologies to enable continuous monitoring during athletic training holds promise in sports cardiology. Future advancements in algorithmic accuracy and robustness could transform early detection of electrolyte disturbances in athletes, provided newer models address current limitations in specificity (especially when it comes to single-lead ECGs) and bias mitigation. Overall, AI-assisted ECG models for electrolyte imbalance detection have demonstrated moderate-to-high diagnostic performance, with AUROC values generally ranging from approximately 0.75 to 0.90 across studied cohorts.

AI-Assisted ECG for the Detection of Aortic Stenosis

The rapid evolution of AI, particularly AI models capable of discerning subtle electrocardiographic (ECG) signatures, has spurred significant interest in their application for identifying aortic stenosis (AS). Pathophysiologically, AS induces structural and functional cardiac alterations such as LVH, diastolic dysfunction, and atrial remodeling. All of which may manifest as distinct ECG patterns. These electrophysiological correlates provide a theoretical foundation for AI-based screening tools, though their clinical translation requires rigorous validation.

The landmark investigation by Kwon et al. pioneered this approach, employing an ensemble architecture to analyze 12-lead and single-lead ECGs from 43,051 patients, including 1,413 (3.3%) with moderate-to-severe AS. In external validation, the model demonstrated robust diagnostic performance, achieving an AUC of 0.86, with sensitivity and specificity of 80% and 78%, respectively [36]. Subsequent studies have sought to refine these methodologies [37-39], with emerging work exploring the feasibility of single-lead ECG analysis to enhance practicality in ambulatory or resource-limited settings [36,40].

Despite these advances, some limitations persist when translating AI-driven ECG analysis into clinical practice. One major issue, pointed out by Gladding and colleagues in their critique of Kwon’s study, is the risk of too many false positives in populations where AS is not that common. In Kwon’s cohort, AS had a prevalence of 3.3%, but the model’s positive predictive value was only 11%. This implies that widespread deployment as a screening tool could precipitate excessive false-positive referrals for echocardiography, straining healthcare systems [41]. This underscores the necessity of context-specific implementation strategies.

Targeted integration of AI-ECG screening may mitigate these limitations. For instance, the selective application of this AI modality in specialized valvular heart disease clinics or cohorts with pathological murmurs. Furthermore, combining AI-ECG analysis with adjunctive modalities such as point-of-care ultrasound may enhance diagnostic yield and streamline triage. Such multimodal approaches align with the growing emphasis on precision cardiology and could prove particularly relevant in sports medicine, where early detection of occult valvular pathology is critical for risk stratification in athletes. AI-assisted ECG models for the detection of AS have demonstrated AUROC values of approximately 0.86 across validation datasets. This discussion naturally leads to the next focus of this review: leveraging AI to optimize echocardiographic acquisition and interpretation.

A summary of diagnostic performance across AI-assisted ECG applications relevant to athlete screening is presented in Table 3.

**Table 3:** Summary of AI-based ECG diagnostic performance across cardiovascular conditions relevant to athlete screening. ML: machine learning; AUROC: area under the receiver operating characteristic curve.

Condition	ECG-based application	Sample size	AI model type	AUROC range	External validation
Left ventricular hypertrophy (LVH)	Structural remodeling detection	66,479	Deep learning/ML	0.85–0.90	Limited
Long QT syndrome (LQTS)	Channelopathy detection	4,521	Deep learning	0.74–0.95	Limited
Electrolyte imbalance	Metabolic disturbance detection	>600,000	Deep learning	0.75–0.90	Variable
Aortic stenosis (AS)	Valvular disease detection	43,051	Ensemble AI models	Approx. 0.86	Yes

AI-assisted physical examination

In the context of athlete pre-participation exams, AI-assisted auscultation has shown significant promise for assisting in detecting and ruling out underlying structural heart disease. Ghanayim et al. [42] integrated a supervised ML algorithm with an AI-enabled stethoscope in 206 patients and reported high diagnostic accuracy for AS. Notably, the algorithm’s sensitivity improved markedly with disease severity, detecting severe AS with 93% sensitivity in severe cases versus 55% in mild cases, with high specificity. Likewise, Prince et al. [43] validated a deep-learning stethoscope algorithm in 615 patients and found that it outperformed clinicians in murmur detection. The AI model reliably identified structural murmurs with an overall sensitivity of 85.6% and specificity of 84.4%, and performance rose to 97.9% sensitivity and 90.6% specificity for clearly audible murmurs in adults. These findings highlight the diagnostic potential of AI-assisted physical exams in athletes: by consistently detecting pathological murmurs and distinguishing them from innocent flow murmurs, such tools can enhance the sensitivity of screening for valvular disease or structural cardiac abnormalities while matching or exceeding clinician accuracy. This improved auscultatory precision is especially critical in sports medicine, where early identification of conditions like valvular stenosis or cardiomyopathic murmurs can guide timely cardiac evaluations and help prevent sudden cardiac events.

AI-assisted transthoracic echocardiography acquisition and analysis

AI-Assisted Image Acquisition

Transthoracic echocardiography (TTE) is essential for diagnosing cardiovascular disease but remains limited by its reliance on skilled sonographers, restricting access in resource-limited and rural settings​ [44]. AI-assisted image acquisition addresses this gap by guiding non-experts to obtain diagnostic-quality images, thereby expanding echocardiography’s reach. The AGILE-Echo trial [44] is a multicenter randomized controlled trial evaluating AI-guided TTE in remote Australian communities, aiming to improve early detection of heart failure and valvular disease in patients who otherwise face delayed diagnoses due to limited specialist availability. Early findings underscore the feasibility of this approach: in one 2024 prospective study, nurses with only brief training, assisted by an AI-guidance system and automated analysis, performed handheld echocardiograms in 13 minutes per patient and accurately identified reduced left ventricular ejection fraction (LVEF < 50%) with an AUC of 0.88 (85% sensitivity, 91% specificity)​ [45]. Such results highlight that AI-enhanced acquisition can “task-shift” echocardiography beyond tertiary centers, enabling effective heart failure screening and general cardiac assessments in primary care, rural clinics, and other low-resource environments. By democratizing image acquisition, AI has the potential to double the yield of asymptomatic cardiac dysfunction detected at the point of care and to initiate timely cardioprotective interventions that would improve outcomes.

AI-Assisted Image Analysis

In parallel, AI is revolutionizing echocardiographic image analysis by automating measurements and interpretation, leading to gains in efficiency and diagnostic consistency. The AI-ECHO randomized crossover trial demonstrated that a fully automated AI analysis platform significantly streamlined workflow: average exam time decreased from 14.3 to 13.0 minutes (p < 0.001) and sonographers’ daily scanning volume increased from 14 to 17 studies (p = 0.003) with AI support​ [46]. Notably, the AI system generated a comprehensive set of 85 quantitative echo parameters per exam (vs. 25 manually) with very high concordance to expert measurements​ [46], allowing more complete assessments (e.g., including advanced metrics like strain) without extra burden​ [47]. Despite handling more cases, sonographers reported significantly less mental fatigue and stress when AI performed the tedious measurements, and blinded observers found that image quality actually improved on AI-assisted days as technicians could focus on probe positioning and patient interaction. Beyond workflow efficiency, AI offers diagnostic speed and breadth: an American Heart Association late-breaking study introduced PanEcho, an AI system capable of interpreting full echocardiographic studies across multiple standard views. PanEcho achieved an average diagnostic AUC of 0.91 across 18 classification tasks and demonstrated near-expert performance in detecting structural and valvular pathology (for example, AUC of 0.95 for left ventricular dilation and 0.99 for severe AS)​ [48]. This comprehensive AI reading markedly reduced the time to results and could expedite care by rapidly flagging critical abnormalities, which is especially valuable in settings lacking on-site cardiologists. Furthermore, large validation trials support that AI maintains diagnostic accuracy: the PROTEUS study (2,341 patients, 20 UK centers) found that an AI decision-support algorithm for stress echocardiography matched expert readers in guiding management and notably improved decision-making for less-experienced clinicians in complex cases​ [49]. Collectively, these advancements illustrate AI’s clinical utility in echocardiography by improving efficiency, standardizing interpretation, and extending high-quality diagnostics to a broader range of clinical environments. The integration of AI-assisted echocardiography in athlete screening programs can significantly enhance the detection of subtle structural abnormalities or valvular pathologies that traditional screening methods might miss. By enabling standardized image acquisition and automated analysis, AI facilitates rapid, consistent, and accurate identification of at-risk athletes, promoting timely intervention and potentially reducing the incidence of sudden cardiac events.

AI-Assisted Coronary Computed Tomography Angiography Analysis

While coronary computed tomography angiography (CCTA) is not typically considered a primary screening tool for asymptomatic athletes, it may serve as a valuable adjunct in cases where ECG and TTE yield inconclusive results. It can also be helpful in ruling out anomalous coronary takeoff, which is linked to sudden cardiac death. Although contemporary CCTA protocols employ dose-reduction techniques and may achieve radiation exposure in the range of approximately 2-5 mSv in selected patients under optimized acquisition protocols, the young age of many athletes necessitates careful consideration of cumulative lifetime radiation risk. Historically, effective radiation doses for CCTA were reported to be substantially higher, with median exposures approaching 12 mSv in earlier multicenter studies, underscoring the importance of judicious imaging selection in younger populations [50,51]. A study by Kübler et al. [52] demonstrated that AI-enhanced CCTA exhibited high sensitivity and negative predictive value (NPV) in detecting significant coronary stenosis, suggesting its potential role in further risk stratification of athletes with borderline assessments. However, it is important to note that the study also reported a lower PPV and a tendency to overestimate disease burden. Emerging AI-based CCTA algorithms have demonstrated the ability to characterize plaque morphology and identify high-risk features such as low-attenuation plaque and positive remodeling, potentially improving risk stratification beyond luminal stenosis assessment and enabling earlier detection of subclinical atherosclerotic disease [53,54]. These findings indicate that while the model requires further refinement, AI-enhanced CCTA holds promise for improving diagnostic accuracy and guiding clinical decision-making, particularly in low-risk populations.

**Figure 1:**
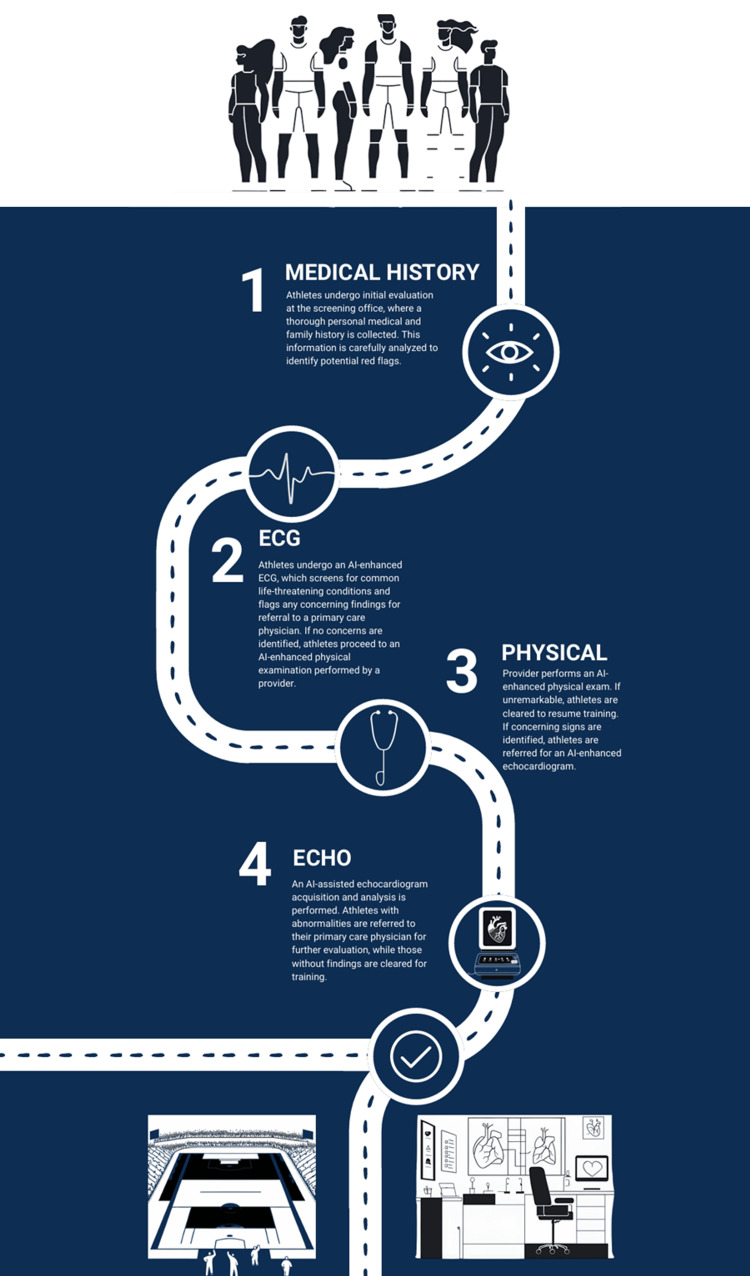
Proposed AI-assisted cardiovascular screening pathway for athletes. This figure is an original illustration created by the authors for the current study using Canva (Sydney, Australia) and Adobe Photoshop (Adobe, San Jose, CA).

Based on the evidence reviewed across electrocardiography, auscultation, echocardiography, and cardiac computed tomography, we propose an integrated AI-assisted cardiovascular screening pathway for athletes. This framework combines structured clinical and family history assessment with AI-enhanced ECG interpretation, AI-supported physical examination, and targeted echocardiographic evaluation to efficiently identify individuals at risk for underlying cardiac pathology. Screening begins with standardized history-taking to identify red-flag symptoms or familial risk, followed by AI-assisted analysis of a 12-lead ECG with human oversight to minimize missed diagnoses. Athletes with reassuring findings proceed to an AI-augmented physical examination, while those with concerning features undergo AI-guided TTE for definitive structural assessment. Normal results allow safe clearance with appropriate follow-up, whereas abnormal findings prompt referral for specialist evaluation and, when indicated, advanced imaging such as coronary CT angiography. A schematic representation of this proposed stepwise AI-assisted screening pathway is presented in Figure 1.

Limitations

Despite the substantial promise of AI in cardiovascular screening, key limitations must be addressed before widespread clinical adoption, particularly in athletic populations. A primary concern lies in the fact that most current AI models have not been trained or validated using datasets derived from healthy athletes. Physiological adaptations common in this group, such as increased ventricular wall thickness or early repolarization patterns, can mimic pathological findings, increasing the risk of false-positive results when algorithms are developed using general clinical cohorts. Such misclassifications may lead to unnecessary downstream testing, inappropriate restriction from sports participation, and increased anxiety for both patients and clinicians. Moreover, the training datasets themselves often lack demographic and ethnic diversity, limiting the external validity and generalizability of these models across broader athletic populations.

Interpretability remains another major barrier. Most DL models lack transparency, offering little insight into the features driving diagnostic predictions. This "black box" nature impedes clinical trust and limits the practical utility of AI tools, especially when nuanced decision-making is required. Compounding this is the current regulatory ambiguity surrounding medical AI. Standardized guidelines for validation, approval, and integration into clinical workflows remain underdeveloped, leaving institutions uncertain about how to responsibly implement these technologies. As the field moves forward, it will be critical to develop athlete-specific training datasets, improve model explainability, and establish rigorous regulatory frameworks. These steps are essential not only to ensure diagnostic accuracy but also to build the clinician's confidence necessary for the meaningful integration of AI into cardiovascular care for athletes.

Based on current evidence, AI-assisted tools should be integrated as decision-support systems rather than standalone diagnostic modalities, particularly in athletic populations where physiological adaptations may confound interpretation.

Future research should focus on prospective validation of AI models in athlete-specific cohorts, development of explainable algorithms, and evaluation of real-world clinical impact on screening outcomes and downstream testing.

## Conclusions

AI demonstrates growing potential to enhance cardiovascular screening in athletes by improving the diagnostic performance and consistency of widely used modalities such as electrocardiography, physical examination, echocardiography, and cardiac computed tomography. When integrated into a structured, multimodal screening framework, AI-assisted tools may support earlier identification of clinically significant cardiovascular pathology while reducing inter-observer variability inherent to traditional interpretation.

A stepwise approach incorporating structured clinical history, AI-assisted ECG analysis, AI-supported physical examination, and targeted imaging may enable more efficient risk stratification and improve the consistency of screening practices across providers involved in athlete evaluation. As further validation studies and athlete-specific datasets emerge, the integration of AI into pre-participation cardiovascular screening workflows may represent a practical strategy to enhance early detection of occult cardiac disease while maintaining scalable and standardized screening processes in athletic populations.
